# Acute Neurological Emergency With Varied Challenges: An Unusual Occurrence and Multimodal Team Approach

**DOI:** 10.7759/cureus.69199

**Published:** 2024-09-11

**Authors:** Sandeep Dey, Ankita Jaiswal, Stuti Bhamri

**Affiliations:** 1 Neuroanesthesiology and Neurocritical Care, Paras Hospital, Gurugram, IND; 2 Neuroanesthesiology and Neurocritical Care, Sarojini Naidu Medical College, Agra, IND; 3 Neuroanesthesiology and Neurocritical Care, National Institute of Mental Health and Neurosciences, Bangalore, IND

**Keywords:** aneurysm coiling, cerebral avm, cerebral vasospasm, microsurgical aneurysm clipping, ruptured cerebral aneurysm, subarachnoid hemorrhage, unruptured cerebral aneurysm

## Abstract

Subarachnoid hemorrhage (SAH) is a devastating condition associated with high mortality and morbidity. Vascular malformations are the most common cause of non-traumatic SAH in patients less than 40 years old. We present a case of a 37-year-old male who presented on the second day of ictus with left-sided hemiparesis and a low Glasgow Coma Scale score (E1VTM5). Non-contrast computed tomography (NCCT) scan of the head was suggestive of right basi-frontal hematoma, SAH, and hydrocephalus (HCP). Given SAH with HCP, the neurosurgical team initially placed a left frontal Ommaya. Cerebral digital subtraction angiography suggested an arteriovenous malformation (AVM) and two anterior cerebral artery aneurysms. Endovascular coiling of the ruptured A2-A3 junction aneurysm was done initially, followed by decompressive craniectomy and evacuation of hematoma and clipping of the still leaky A2-A3 junction aneurysm, also on the same day. The patient recovered in the intensive care unit and was discharged home in good health on the 18th postoperative day. Our case report presents the unique challenge of neuroprotection and maintaining intra-cerebral dynamics in a patient with cerebral aneurysms, AVM, SAH, and hematoma between coagulation (to prevent intra-cerebral hemorrhage) versus anti-coagulation (to prevent emboli during coiling), hypertensive therapy (to prevent cerebral vasospasm) versus relative normotension (to prevent rebleed), and early intervention (surgery and coiling) versus staged procedure. Our multimodal team approach was highly effective in successfully managing the patient and thus highlights its role in managing such critically ill patients.

## Introduction

Subarachnoid hemorrhage (SAH) is a devastating condition associated with high mortality and morbidity [1]. Vascular malformations are the leading cause of non-traumatic SAH in patients under 40 years, with aneurysms accounting for 80-85% and arteriovenous malformations (AVMs) accounting for another 5% [2]. Studies and meta-analyses have reported an incidence of 2.7-18% of intracranial aneurysms associated with AVM [3,4]. The presence of both aneurysm and AVM, though not uncommon, is an essential aspect of treatment decision-making as both have different management strategies, as well as overall prognosis and functional outcome, if not treated in time. This case report emphasizes the significance of a collaborative approach involving different medical specialties in treating patients with high-grade aneurysmal subarachnoid hemorrhage (aSAH) and concurrent intracerebral AVM, along with other poor prognostic indicators. The result of the coordinated, prompt, and evidence-based teamwork was a patient who recovered neurologically, experienced significantly reduced morbidity, and was discharged home with good functional recovery.

## Case presentation

A 37-year-old male presented with sudden onset severe headache for two days, followed by loss of consciousness since early morning on the day of presentation. There was a history of occasional alcohol intake, with no history of trauma, seizure, fever, prior medical illness, any known comorbidity, smoking, or tobacco intake. The patient was taken to a nearby hospital, intubated, and referred to a higher institute due to a low Glasgow Coma Scale (GCS) score. Upon arrival in the emergency room (ER), the patient was unconscious, with decreased movement of the left upper and lower limbs, on mechanical ventilatory support (GCS: E1VTM4), and with the following vitals: blood pressure (BP) = 174/80 mmHg, pulse rate = 98/min, oxygen saturation (SpO2) = 99% (with 50% fraction of inspired oxygen (FiO2) and volume-controlled mode of ventilation), and pupils were bilaterally equal and sluggishly reacting to light.

**Figure 1:**
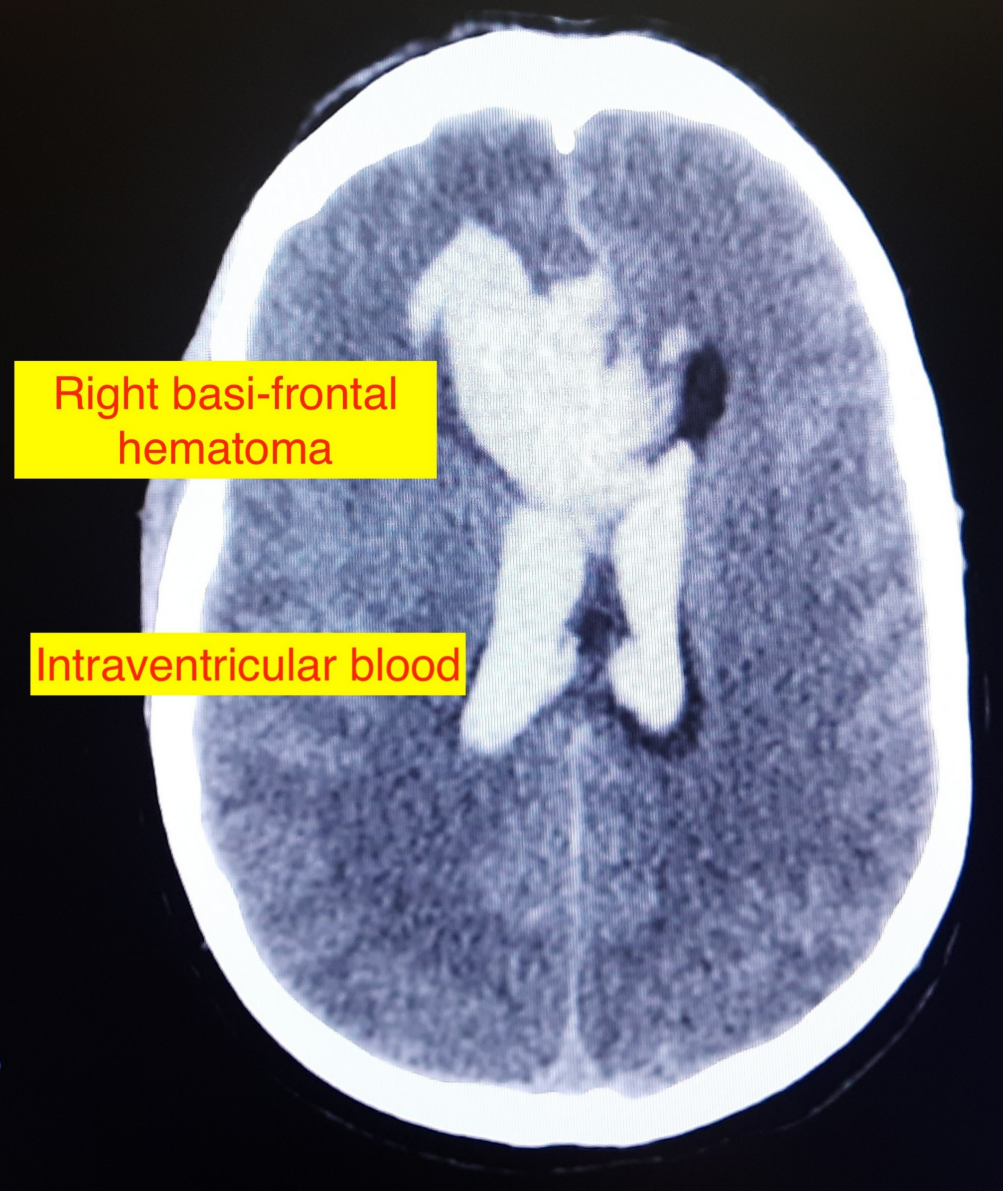
Non-contrast computed tomography of the head showing right basi-frontal hematoma with intraventricular extension.

An immediate CT scan of the head was consistent with acute hematoma (4.1 x 3.9 cm) in the right basi-frontal lobe with intraventricular extension and midline shift of 6 mm toward the left side, mild SAH, a thin subdural hematoma within the anterior falx, and hydrocephalus (Figure 1). CT angiography (CTA) done outside suggested a 5 × 4 mm saccular aneurysm arising from the A2 segment of the right anterior cerebral artery (ACA). There was a 4 × 3 × 2.9 cm large nidus of malformed vessels in the right high parietal region from branches of the right ACA and middle cerebral artery (MCA) and venous drainage into cortical veins on the right side and superior sagittal sinus, suggestive of AVM. Also, there was a 58 × 44 × 41 mm large acute hematoma in the right basi-frontal region with intraventricular extension and acute SAH on the bilateral Sylvian and frontotemporal region. Based on the above findings, we classified the patient as high-grade aSAH with modified Hunt and Hess grade IV, modified Fisher scale grade II, and World Federation of Neurosurgical Societies (WFNS) grade V.

**Figure 2:**
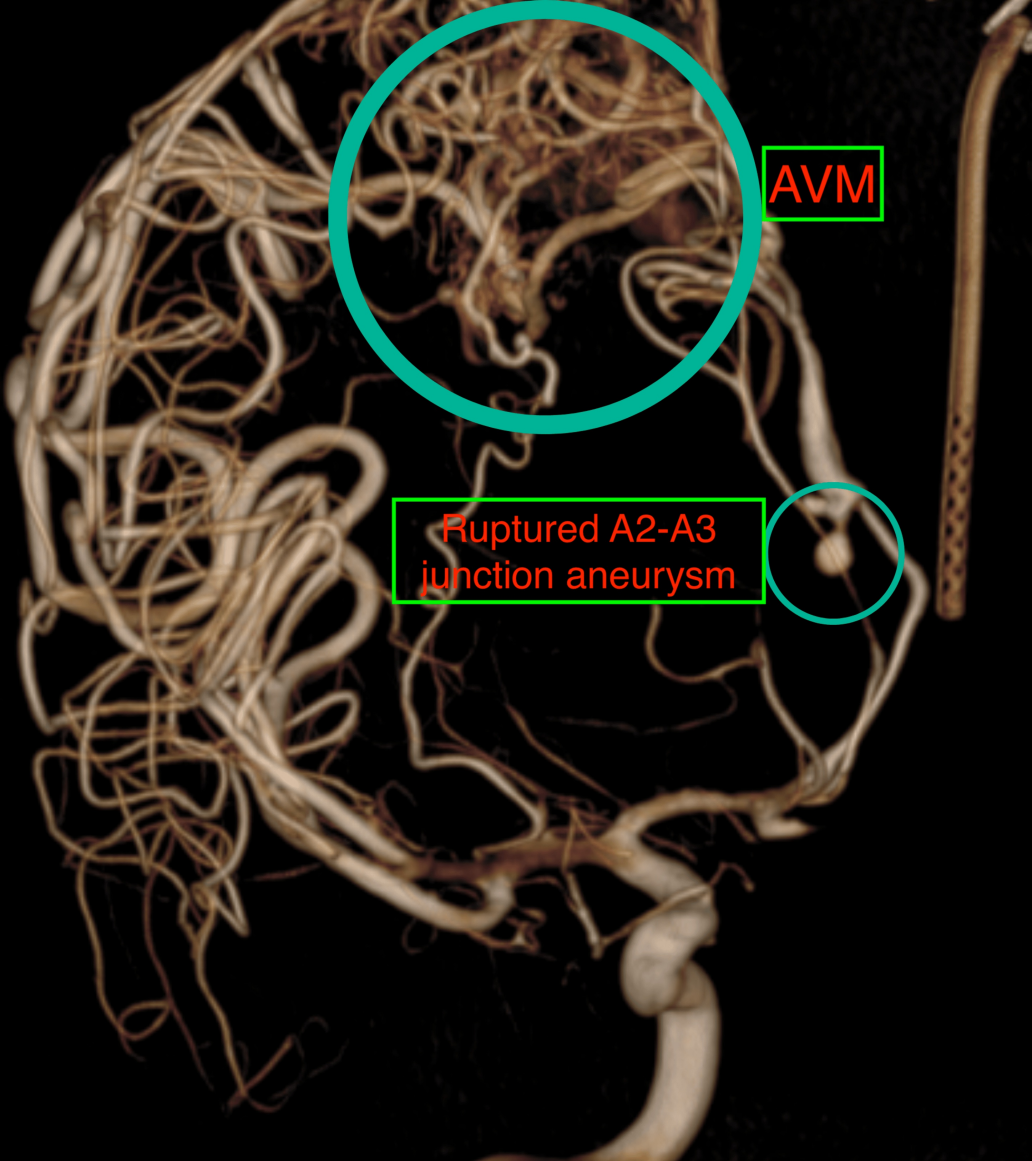
Cerebral DSA showing AVM and A2-A3 junction ruptured aneurysm of the right ACA. DSA, digital subtraction angiography; AVM, arteriovenous malformation; ACA, anterior cerebral artery.

**Figure 3:**
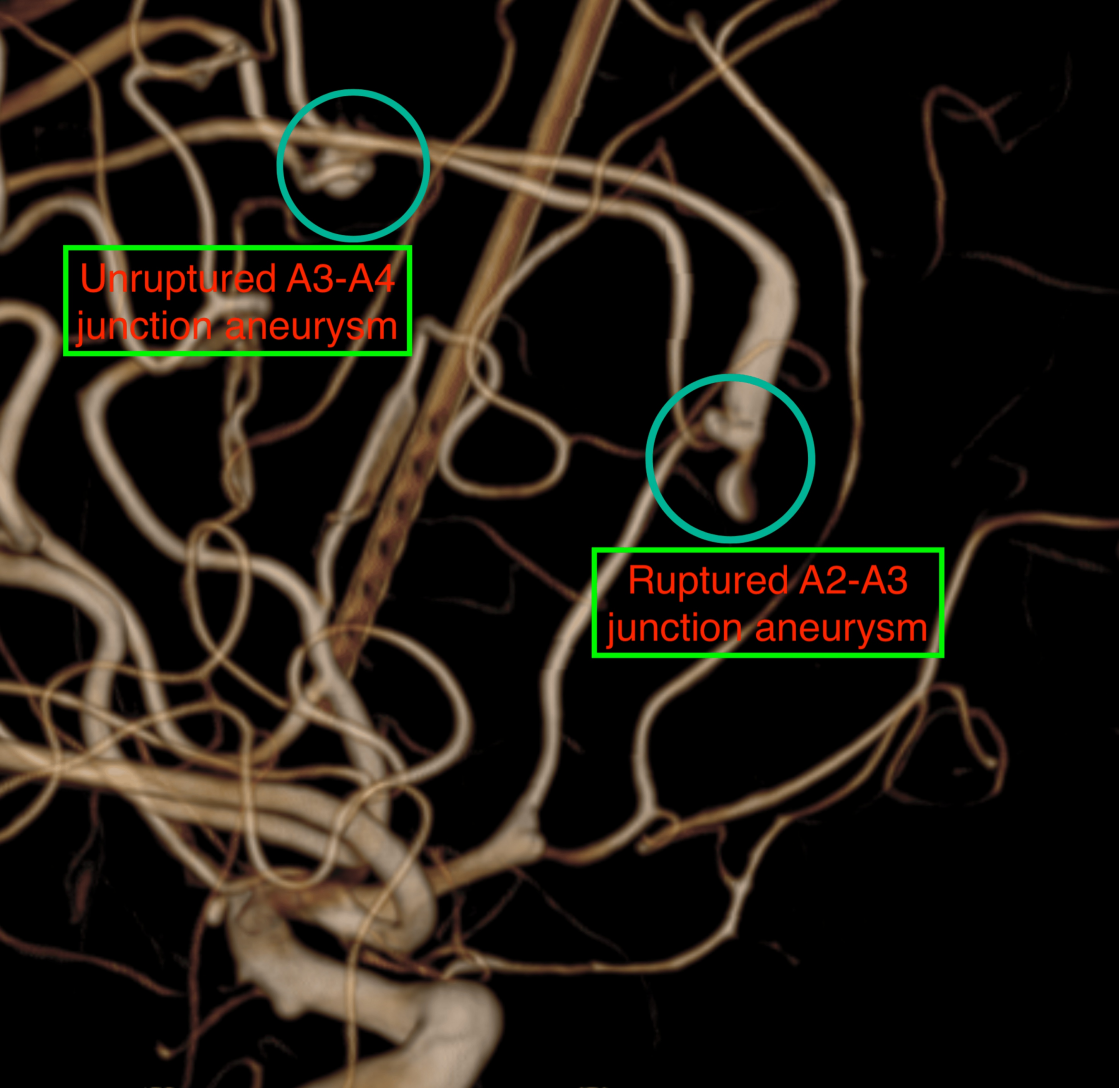
Cerebral DSA showing ruptured A2-A3 and unruptured A3-A4 junction aneurysms of the right ACA. DSA, digital subtraction angiography; ACA, anterior cerebral artery.

Our initial evaluation suggested multiple pathologies, including AVM, flow-related aneurysms, SAH, hematoma with mid-line shift, and hydrocephalus, each with a different threat to life and functional outcome. Because of SAH with hydrocephalus and the necessity of anti-coagulation during the endovascular procedure, the patient underwent an initial left-sided frontal Ommaya placement within an hour of presentation to the ER. We secured an arterial line and a central venous catheter apart from the standard monitors during the procedure. We maintained anesthesia during this period using oxygen and air (with 50% FiO2), 1% propofol target-controlled infusion (TCI), fentanyl (1 mcg/kg/hour), and atracurium (0.5 mg/kg/hour) infusions, and labetalol infusion to maintain systolic BP around 140 mmHg while avoiding large fluctuations and variability. We used a balanced salt solution to provide hydration. Following Ommaya reservoir placement, the neurointervention team went ahead with a four-vessel cerebral digital subtraction angiography (DSA) to delineate the vascular malformations and define treatment options. On the right internal carotid artery injection, there was a right posterior frontal AVM with an aneurysm at the A2-A3 junction, measuring 2.4 x 3.5 x 3.2 mm with a narrow neck, directed inferiorly and laterally, and a second unruptured A3-A4 junction aneurysm, measuring 3.5 x 2.4 x 3.2 mm, directed superiorly and laterally (Figures 2, 3).

**Figure 4:**
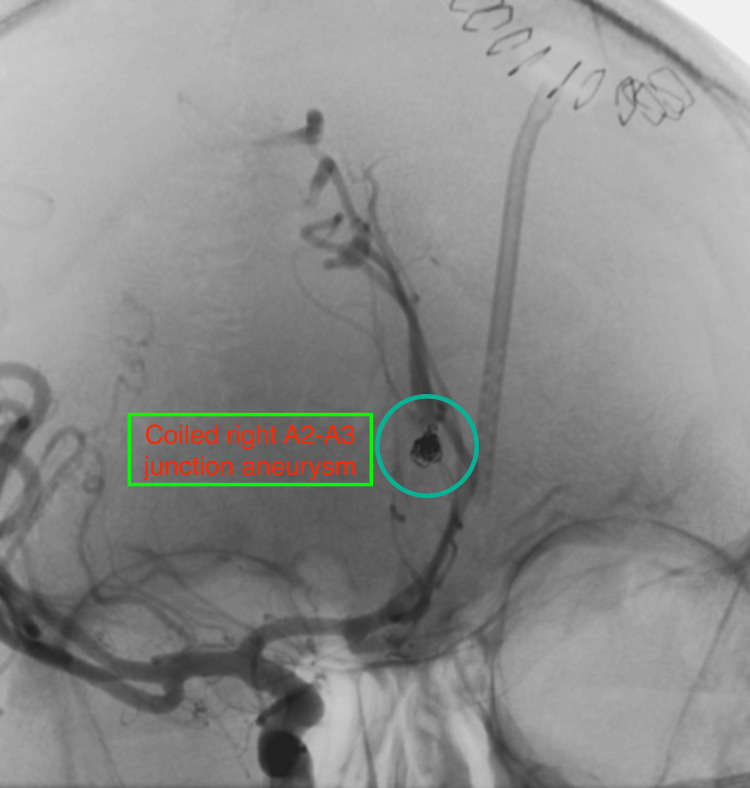
Cerebral DSA showing coiled A2-A3 junction aneurysm of the right ACA. DSA, digital subtraction angiography; ACA, anterior cerebral artery.

After thorough consideration, the intervention team decided to do endovascular coiling of the ruptured right A2-A3 junction aneurysm the same day. During the procedure, we maintained anesthesia and mechanical ventilation with no positive end-expiratory pressure. We continued the balanced salt solution and titrated the rate to keep the systolic pressure variation at around 10. We maintained mild hypocapnia, normoglycemia, normothermia, and normal acid-base balance through serial arterial blood gas (ABG) analysis. We maintained anti-coagulation with heparin boluses to keep the activated clotting time (ACT) two to three times above the baseline. Using labetalol infusion, we controlled the mean arterial pressure (MAP) at around 70-80 mmHg to prevent abrupt changes in transmural pressure that can lead to aneurysm sac rupture. For endovascular coiling, the neuro-interventional team gained access to the aneurysm using a microcatheter and placed a few detachable coils in the sac (Figure 4). Check DSA suggested good antegrade flow in distal ACA with no flow in the aneurysm sac. Heparin was reversed toward the end of the procedure using protamine, repeated at intervals, till the return of ACT to baseline. CT scan of the brain done post-procedure did not suggest any increase in bleeding. After that, we titrated the labetalol infusion to a target MAP of 80-90 mmHg. The patient was kept sedated, paralyzed, and ventilated, along with nimodipine infusion (1 mg/hour) to prevent cerebral vasospasm.

**Figure 5:**
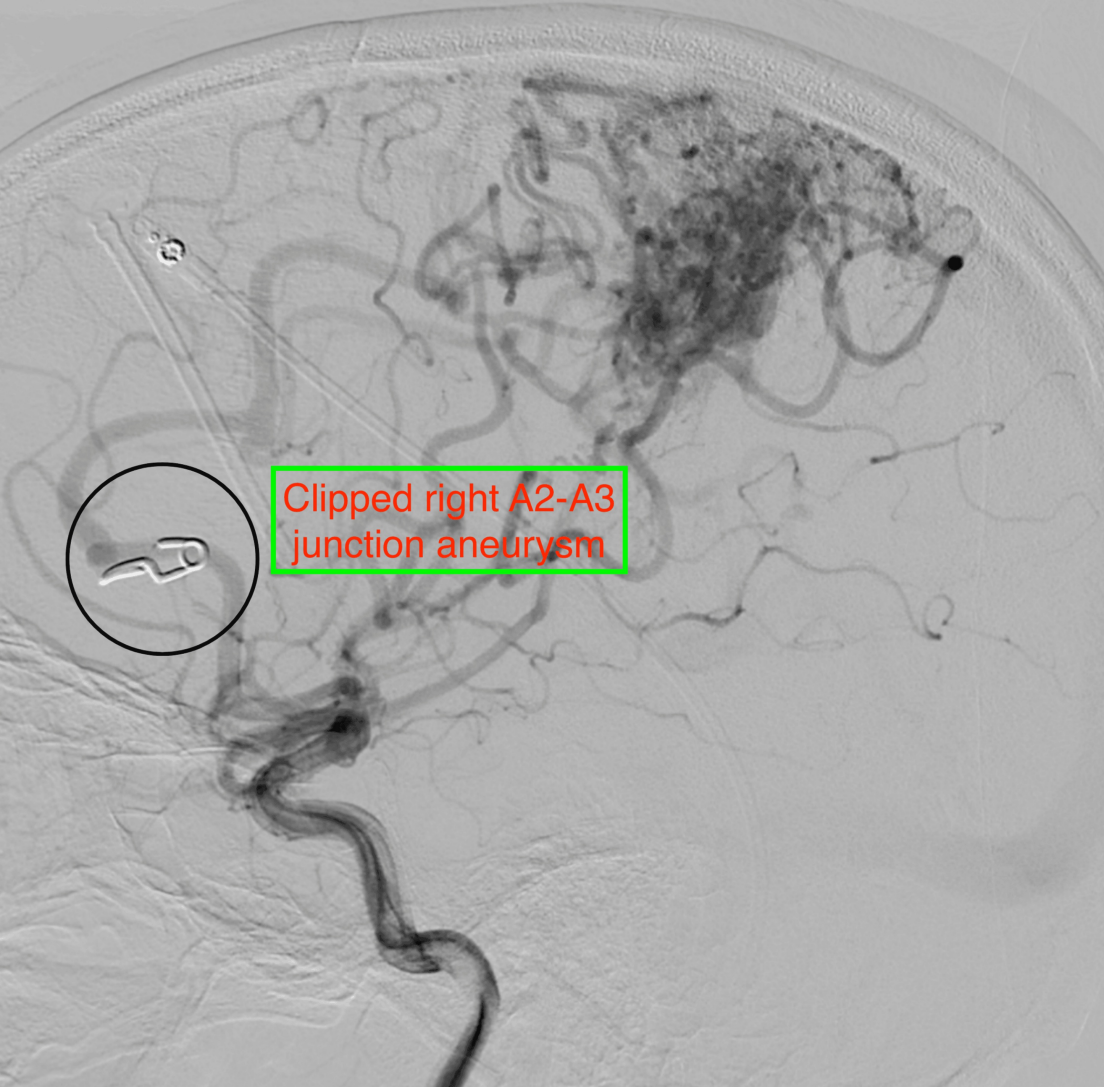
Cerebral DSA showing clipped A2-A3 junction aneurysm of the right ACA. DSA, digital subtraction angiography; ACA, anterior cerebral artery.

Given the large frontal hematoma with midline shift and altered sensorium, the neurosurgical team decided on an emergency right frontotemporoparietal decompressive craniectomy and evacuation of the hematoma. During hematoma evacuation, the A2-A3 junction aneurysm was found to ooze and, hence, it was also clipped to prevent the risk of rebleeding (Figure 5). We continued our anesthetic regimen as prior, titrated the MAP between 80 and 90 mmHg, and added 20% 20M mannitol for brain relaxation. The procedure was undertaken under the cover of fresh frozen plasma (FFP) and packed red blood cells (PRBC) because of the ongoing ooze and blood loss.

After the procedure, it was decided to keep the patient sedated, relaxed, and ventilated for two days. We used infusions of fentanyl (1 mcg/kg/hour), midazolam (0.1 mg/kg/hour), propofol (50 mcg/kg/min), and atracurium (0.1 mg/kg/hour). At the same time, a nimodipine tablet was given via a Ryles tube to prevent vasospasm. Serial ABG, electrolyte profile, chest X-ray, non-contrast computed tomography (NCCT) of the head, transcranial Doppler (TCD), and other standard monitoring were done per institutional protocol, and necessary corrections were made.

**Figure 6:**
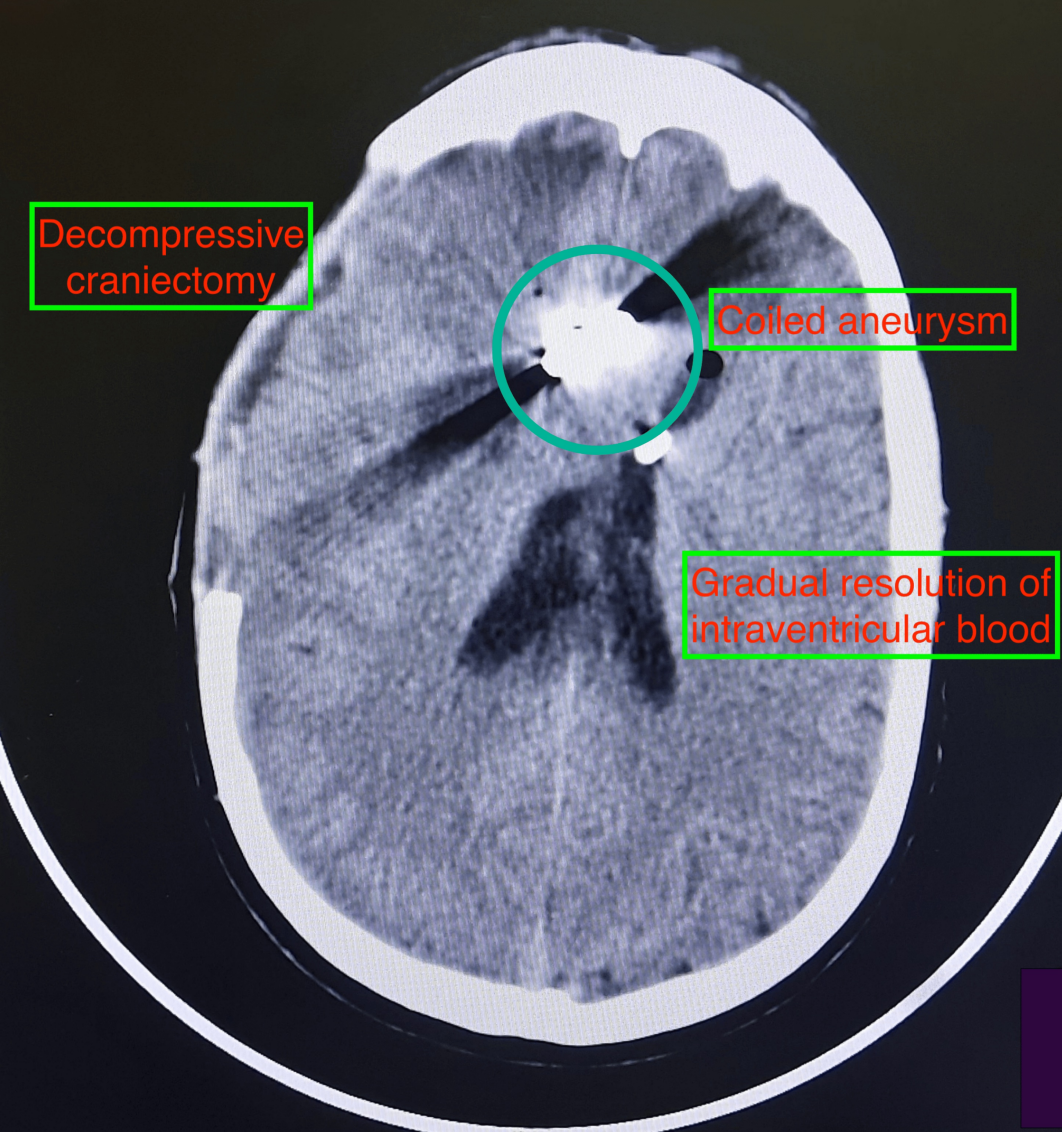
Non-contrast computed tomography of the brain showing coiled aneurysm, decompressive craniotomy, and gradual resolution of intraventricular blood.

On postoperative day (POD) two, we decided to give the patient a weaning trial. All infusions were stopped, and reversal was done for the residual effect of the muscle relaxant. We did a slow and gradual weaning until the patient maintained respiration with minimal pressure support. A repeat NCCT of the head revealed the ongoing resolution of intraventricular and intraparenchymal blood (Figure 6). The patient maintained his vitals with gradual improvement in neurological and respiratory parameters, and we eventually extubated the patient. The post-extubation phase was uneventful, with a GCS of E4V3M6. We kept the patient nil orally and maintained hydration with a balanced salt solution. All routine care was continued, including physiotherapy and intermittent pneumatic compression for deep vein thrombosis prophylaxis.

By POD three, Ryles tube feeding was started and titrated to provide a protein and calorie supplementation of around 2 g/kg/day and 25-30 Kcal/kg/day, respectively. Hemoglobin, electrolytes, and albumin were routinely assessed and corrected as necessary. By POD four, the patient had a GCS of E4V4M6, and the external ventricular drain (EVD) was removed, as the CSF output was non-bloody, there were no signs of raised intracerebral pressure, and the patient was neurologically stable. We continued regular neurological assessments, including TCD, to watch for cerebral vasospasm. By POD six, there was gradual improvement in the left-sided motor weakness, and the physiotherapy team could mobilize the patient out of his bed with support. On POD 12, the patient underwent a left-sided ventriculoperitoneal shunt for hydrocephalus secondary to intraventricular bleed. The patient was shifted to the neurosurgical ward, where he had a stable course and good functional recovery. On POD 18, the patient was discharged home, with advice to follow up for definitive treatment of the cerebral AVM.

## Discussion

Cerebral aneurysms are the most common vascular malformations associated with non-traumatic SAH [2]. Aneurysm may be associated with AVM in 2.7-18% of cases [3,4]. Cerebral aneurysms associated with AVM can be intranidal, flow-related, and unrelated [5]. The coexistence of aneurysms with AVM increases the relative risk of bleeding at presentation and is associated with a worse natural history than alone [6]. Flow-related aneurysms are rare and may be located on the wall of a feeding artery (arterial aneurysm) or draining vein (venous varices). Pathophysiology of flow-related aneurysms is complex and thought to be predisposed by flow (hemodynamic) factors, patient-specific factors (smoking, alcohol), or genetics. Hemorrhagic manifestations are common with intranidal AVMs [5]. Also, it has been reported that aneurysms are common with AVMs located in the infratentorial compartments [7].

Currently, no consensus guideline exists on treating intracranial aneurysms associated with AVM. In a systemic review and meta-analysis of ruptured and unruptured aneurysms, treating AVM has been associated with a complication rate of 0-63% for radiofrequency ablation, 1.5-54% for microsurgery, and 7.6-55% for endovascular embolization [8]. Treatment of intracranial AVM with an aneurysm is dependent upon their anatomical relationship (proximal or distal), anatomical integrity (ruptured or unruptured), site of rupture, source of SAH and or intracerebral hematoma (AVM or aneurysm), the weighted risk-benefit ratio, availability of microsurgical or endovascular or radiological modality, and individual as well as institutional expertise. Based on the above, there can be three case scenarios: (1) cases with bleeding secondary to an AVM or intranidal aneurysm are associated with a low rebleed rate. Conservative management can be the initial approach, followed by definitive treatment based on risk stratification. (2) Distal flow-related aneurysms are associated with a high spontaneous remission rate (up to 80%), with no rebleed on long-term follow-up following complete occlusion of AVM nidus [9]. Hence, small distal flow-related unruptured aneurysms are mostly treated conservatively following definitive treatment of unruptured AVM nidus. (3) When bleeding is secondary to an aneurysm, management is along the lines of an isolated ruptured aneurysm [6,10].

Treatment of the associated unruptured AVM can be postponed, with the crux of aneurysm recurrence, until definitive obliteration of the nidus.

Aneurysmal SAH is a life-threatening condition. Early diagnosis and treatment (preferably within 24 hours of onset of SAH) improves outcome, reduces risk of rebleed, and facilitates treatment of delayed cerebral ischemia (DCI). An NCCT of the head is the initial investigation done in patients presenting with acute onset severe nontraumatic thunderclap headaches. CT angiography or cerebral DSA confirms the diagnosis. Cerebral DSA is also beneficial if CTA is negative or inconclusive for aSAH with high suspicion and in diffuse SAH. Also, DSA can define treatment strategy. Following the initial radiodiagnosis, one should control BP with short-acting antihypertensive agents. The recent guideline recommends a gradual reduction of severely elevated BP (systolic BP > 180-200 mmHg) while avoiding hypotension (MAP < 65 mmHg) [11]. Systolic BP > 160 mmHg is associated with higher rates of rebleed [11]. Treatment of aSAH is a multidisciplinary team approach. Definitive management of the ruptured aneurysm by surgical clipping or endovascular coiling is done as soon as possible after presentation, after weighing the risk-benefit of either procedure in an individual patient and the aneurysm characteristics. In patients with good-grade SAH, endovascular coiling has been associated with better functional independence at one year compared to surgical clipping [12,13]. Similarly, endovascular coiling is preferred in patients with high-grade SAH, although there is a lack of definitive evidence-based data [11]. In patients with a depressed level of consciousness secondary to a large intra-parenchyma hematoma, emergent clot evacuation has been shown to have a mortality benefit [14].

The goals of anesthetic management during surgical or endovascular treatment of aSAH are to provide a stable hemodynamic, favorable ventilation, and patient immobility. In the presence of neurological impairment, it is preferable to go for general endotracheal anesthesia, using continuous infusions to maintain a stable state of hypnosis, analgesia, amnesia, and muscle relaxation. Isotonic solutions are used for fluid management, avoiding hypotonic fluids. Hyperosmotic solutions (mannitol or hypertonic saline) provide brain relaxation, decrease intracerebral pressure (ICP), and improve cerebral blood flow. Poor glycemic control in the perioperative period increases the risk of worse clinical outcomes. There is no consensus defining optimal intra-operative volume status; however, both hypovolemia (leading to DCI) and hypervolemia (leading to respiratory complication) have detrimental effects [11]. Recent guidelines do not recommend any specific BP target during elevated ICP, acute aneurysm rupture, or the intra-operative period. However, it stresses frequent BP monitoring and maintaining stable hemodynamics to prevent ischemia or re-rupture [11]. Also, the current guideline states no consensus on the therapeutic benefit of systemic hypothermia; on the contrary, hyperthermia can predispose to poor perioperative outcomes [11]. There is no set protocol for peri-procedural anesthetic management, and it is mostly institutionally based. Regarding supportive care, the optimal method for continuous intravascular volume status measurement is still controversial. Early goal-directed fluid therapy using stroke volume variability or cardiac output better detects intravascular volume status than the conventional methods [11]. Prophylactic-induced hypervolemia leads to increased medical complications with no impact on outcome [11]. Venous thromboembolism (VTE) complicates around 4-24% of patients with acute aSAH [15], and thromboprophylaxis using mechanical devices or pharmacological agents can decrease the VTE rate. The optimal timing to start VTE prophylaxis post-procedure or surgery is not yet defined [11]. Perioperative hyperglycemia and fever are independently associated with unfavorable outcomes following aSAH [11]. Both hyperglycemia and hypoglycemia can lead to secondary brain injury. However, the current guideline has not defined the target glycemic threshold and treatment intensity (conventional versus intensive). aSAH is a catabolic state, and early nutrition support improves outcomes in select patient groups [11]. In patients with good grade SAH, frequent assessments of vitals and neurological status are recommended using validated tools like GCS, National Institutes of Health Stroke Scale (NIHSS), or VASOGRADE to monitor neurological recovery and DCI as well as any secondary cerebral insult [11]. Neurogenic dysphagia can develop in up to 65% of patients following aSAH [16]. Dysphagia screening done before initiating feeding by mouth prevents pneumonia and long-term mortality. In patients with secured aneurysms, early mobilization can prevent VTE, pressure sores, pneumonia, and vasospasm and is associated with good functional recovery [17]. Around 30% of patients with aSAH can develop DCI. TCD serves as a bedside tool to screen for vasospasm. It is non-invasive, valid, sensitive, and particularly helpful in patients with high-grade SAH and on mechanical ventilation, which limits neurological assessment [11]. Enteral nimodipine is an essential add-on in the postoperative armamentarium to prevent DCI. Similarly, maintaining euvolemia has been found to avoid vasospasm, improve functional outcomes, and reduce cardiac and pulmonary complications post aSAH [11]. BP variability is unfavorable, and hypotension precedes neurological deterioration. Hemodynamic augmentation (intravenous colloids, vasopressors) improves neurological and functional outcomes following DCI. Permissive autoregulation has the same neurological outcome as prophylactic augmentation but with fewer complications. aSAH can lead to acute symptomatic hydrocephalus (estimated incidence: 15-87%) [18]. Urgent CSF diversion (EVD or lumbar drain) improves neurological recovery and prevents DCI. Moreover, 8.9-48% of patients can develop chronic shunt-dependent hydrocephalus, requiring permanent CSF diversion [18]. Patients with Hunt and Hess grade > 3, Fisher grade III/IV, and hydrocephalus are at elevated risk of seizure, and anti-seizure prophylaxis is reasonable [19]. Incomplete occlusion of ruptured aneurysms poses a high risk of re-rupture and requires immediate intervention [20].

## Conclusions

Aneurysms are associated with high mortality and morbidity. It should be treated with surgical clipping or endovascular coiling, as per the merit of the case, and as early as possible, unless otherwise contraindicated. Leak following endovascular coiling (endo leak) may not be apparent on an immediate cerebral DSA and will be clinically evident only later with substantial mass effect in patients who are sedated and mechanically ventilated. An integrated approach and timely management of such vascular malformations is not only lifesaving but also reduces the overall morbidity, cost of treatment, and length of hospital stay.
